# Non-canonical deubiquitination of OTUB1 induces IFNγ-mediated cell cycle arrest via regulation of p27 stability

**DOI:** 10.1038/s41388-024-03042-z

**Published:** 2024-04-25

**Authors:** Seul Gi Lee, Seon Min Woo, Seung Un Seo, Hyun Shik Lee, Sang Hyun Kim, Young-Chae Chang, Hyo Je Cho, Simmyung Yook, Ju-Ock Nam, Taeg Kyu Kwon

**Affiliations:** 1 Department of Immunology, School of Medicine, Keimyung University, Daegu, 42601 South Korea; 2 Department of Microbiology, Immunology, and Cancer Biology, University of Virginia, Charlottesville, VA 22908 USA; 3 Center for Forensic Pharmaceutical Science, Keimyung University, Daegu, 42601 South Korea; 4 School of Life Sciences, BK21 Plus KNU Creative BioResearch Group, College of Natural Sciences, Kyungpook National University, Daegu, 41566 South Korea; 5 Department of Pharmacology, School of Medicine, Kyungpook National University, Daegu, 41944 South Korea; 6 Research Institute of Biomedical Engineering and Department of Medicine, Catholic University of Daegu School of Medicine, Daegu, 42472 South Korea; 7 Department of Biochemistry, Chungbuk National University, Cheongju, 28644 South Korea; 8 Department of Biopharmaceutical Convergence, Sungkyunkwan University, Suwon, 16419 South Korea; 9 Department of Food Science and Biotechnology, Kyungpook National University, Daegu, 41566 South Korea

**Keywords:** Ubiquitylation, Growth factor signalling, Breast cancer

## Abstract

The deubiquitinase OTUB1, implicated as a potential oncogene in various tumors, lacks clarity in its regulatory mechanism in tumor progression. Our study investigated the effects and underlying mechanisms of OTUB1 on the breast cancer cell cycle and proliferation in IFNγ stimulation. Loss of OTUB1 abrogated IFNγ-induced cell cycle arrest by regulating p27 protein expression, whereas OTUB1 overexpression significantly enhanced p27 expression even without IFNγ treatment. Tyr26 phosphorylation residue of OTUB1 directly bound to p27, modulating its post-translational expression. Furthermore, we identified crucial lysine residues (K134, K153, and K163) for p27 ubiquitination. Src downregulation reduced OTUB1 and p27 expression, suggesting that IFNγ-induced cell cycle arrest is mediated by the Src-OTUB1-p27 signaling pathway. Our findings highlight the pivotal role of OTUB1 in IFNγ-induced p27 expression and cell cycle arrest, offering therapeutic implications.

## Introduction

OTUB1, a deubiquitinating enzyme (DUB) in the OTU-containing protein family, is predominantly expressed in various tumor types [1, 2]. It has been reported that OTUB1 acts either as a conventional DUB by directly cleaving ubiquitin chains or hinders substrate ubiquitination through non-canonical interference with the activities of E2 [1, 3, 4]. Notably, the non-canonical pathway operates independently of catalysis, with studies indicating that OTUB1’s deubiquitination in this mode affects protein stability by sequestering E2 enzymes from target protein substrates [5, 6]. Recent findings highlight the inhibitory effect of non-canonical OTUB1 activation on HIF-1α protein degradation, resulting in cellular metabolic adaptation and hypoxia resistance [7]. Our previous research demonstrated that OTUB1 regulates Raptor stability through E2 enzyme-mediated ubiquitination in a non-canonical manner [2]. This non-canonical pathway is closely related to the response of tumor cells to antitumor agents. However, the full scope of the additional biological functions governed by non-canonical OTUB1 remains largely unexplored.

Research has consistently demonstrated the pro-apoptotic effects of Interferon‐gamma (IFNγ) on various tumor cell types [8, 9]. However, conflicting findings exist regarding its overall biological impact on tumors [10–12]. IFNγ induces cell cycle arrest and apoptosis in tumor cells via the JAK-STAT-caspase pathway [13]. For instance, IFNγ treatment selectively induces pro-apoptotic effects in tumor-initiating colon cancer cells [14]. Conversely, low-dose IFNγ treatment facilitates stem-like properties through the ICAM1-PI3K-Akt-Notch1 pathway, ultimately fostering tumor growth [15]. Despite extensive investigation into the IFNγ signaling pathway, the precise regulatory mechanisms governing IFNγ signaling and its impact on cell cycle arrest remain unclear. p27 has a pivotal role in mediating IFNγ-induced cell cycle arrest [16]. The Skp2/p27 signaling axis plays a dual role in IFNγ-induced tumor growth inhibition and G1 arrest in melanoma cells [17]. Multiple studies have been reported that p27 expression and function is predominantly through post-transcriptional level, both at translation and degradation stages [18, 19].

In this study, we investigated the impact of OTUB1 on IFNγ-induced cell cycle arrest and its regulatory mechanism. OTUB1 deletion hindered IFNγ-mediated cell cycle arrest by downregulating p27 protein expression. Furthermore, we identified a non-canonical modulation of OTUB1-mediated p27 protein stability. The phosphorylation of OTUB1 at Tyr26 emerged as a critical factor in IFNγ-induced p27 upregulation.

## Results

### IFNγ upregulates p27 expression and OTUB1 phosphorylation at Tyr26

**Fig. 1:**
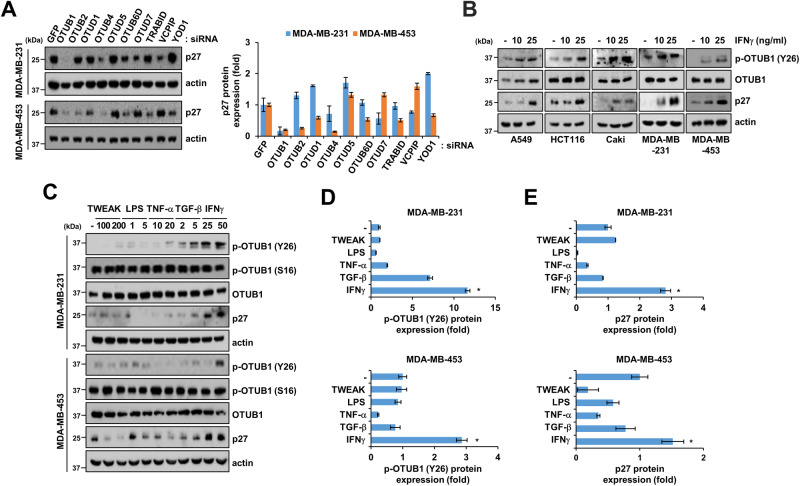
IFNγ induces phosphorylation of OTUB1 Tyr26 and p27 expression in various cancer cells. **A** MDA-MB-231 and MDA-MB-453 were transfected with DUB siRNA of OTU family. p27 protein expressions were verified through western blotting and quantified. **B** A549, HCT116, Caki, MDA-MB-231, and MDA-MB-453 cancer cells were incubated in serum-free media for 12 h and treated with IFNγ (10–25 ng/mL) for 30 h. **C–E** MDA-MB-231 and MDA-MB-453 cells were stimulated with indicated cytokines for 30 h, and protein expression was determined. Actin served as the loading control. *P < 0.01 compared to control.

To understand the impact of DUBs on p27 expression, we investigated the OTU family of DUBs and their regulation of p27 protein expression. Among the tested DUBs, silencing OTUB1 with siRNA consistently reduced p27 expression in both MDA-MB-231 and MDA-MB-453 cells (Fig. 1A). Furthermore, consistent with previous findings, IFNγ treatment elevated p27 expression across various cancer cell types, including A549, HCT116, Caki, MDA-MB-231, and MDA-MB-453. The dose-dependent increase in phosphorylation at Tyr26 of OTUB1 and p27 expression was evident with IFNγ treatment (Fig. 1B). Additionally, we assessed the effects of other cytokines, such as TWEAK, LPS, TNF-α, and TGF-β, on OTUB1 phosphorylation (Tyr26) and p27 expression. While TNF-α marginally increased p-OTUB1 (Tyr26) and p27, our finding highlights IFNγ as the superior cytokine, distinctly upregulating the expression of these proteins in MDA-MB-231 and MDA-MB-453 cells compared to all other cytokines tested in this study (Fig. 1C–E).

### IFNγ augments G0/G1 population

**Fig. 2:**
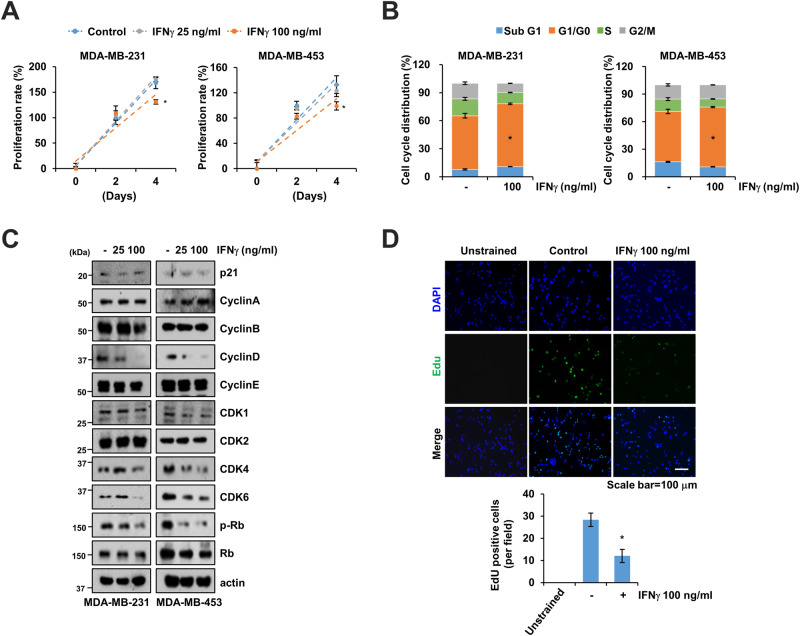
IFNγ induces G0/G1 cell cycle arrest in breast cancer cells. **A** MDA-MB-231 and MDA-MB-453 cells were treated with IFNγ (25–100 ng/mL) for 4 d. Cell viability was verified using MTT assay at the indicated time points. **B**, **C** IFNγ-treated cells were subjected to cell cycle analysis and related protein expression using FACS and western blotting, respectively. **D** Representative EdU staining (green) and DAPI (blue) images of MDA-MB-231 cancer cells. Scale bar = 100 μm. Error bars represent mean ± SEM. *P < 0.01 compared to control.

To investigate the functional role of IFNγ, we assessed its impact on cell proliferation and cell cycle distribution. IFNγ treatment at a concentration of 100 ng/mL significantly inhibited cell growth and induced G0/G1 cell cycle arrest in MDA-MB-231 and MDA-MB-453 cells (Fig. 2A, B). IFNγ treatment led to a dose-dependent decrease in cyclin A, CDK4, CDK6, and p-Rb expression while exhibiting opposing effects on p27 expression (Figs. 2C and 1B). No significant differences were observed in the expression of p21, cyclin A, cyclin B, cyclin E, CDK2, or Rb (Fig. 2C). Additionally, IFNγ treatment reduced proliferative cells labeled with the proliferation marker EdU (Fig. 2D). Furthermore, we examined the impact of IFNγ on ERα-positive breast cancer cells MCF-7 and MDA-MB-361. There cells exhibited a slight response to IFNγ challenge with minor changes in p-OTUB1 (Tyr26) and p27 expression, as well as cell cycle arrest compared to triple negative breast cancer cells MDA-MB-231 and MDA-MB-453 (Supplementary Fig. 1A, B).

### OTUB1 knockdown suppresses IFNγ-induced post-translational p27 expression

**Fig. 3:**
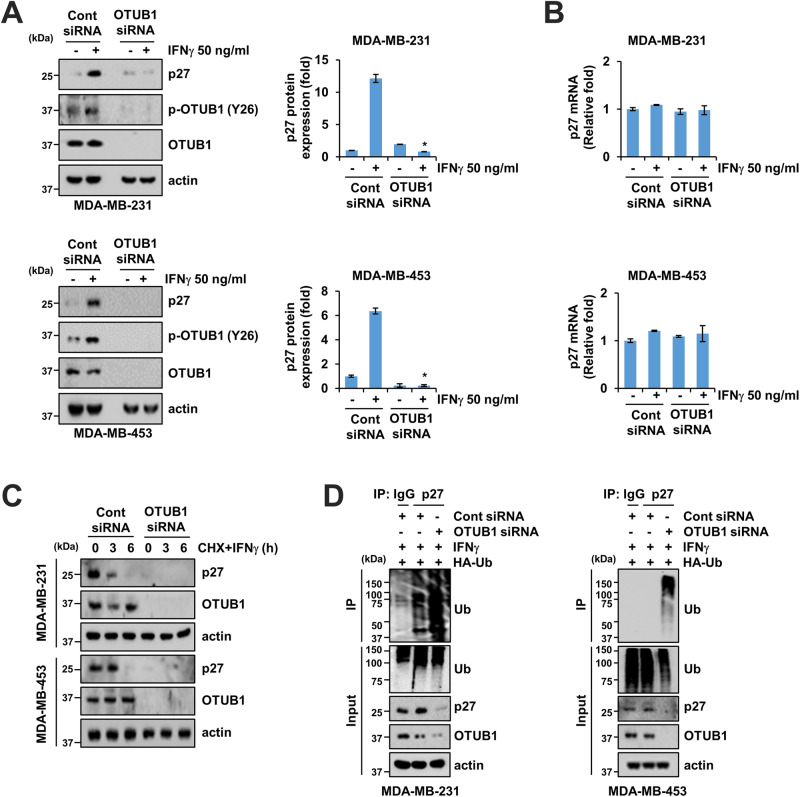
OTUB1 knockdown promotes post-translational degradation of p27. **A**, **B** MDA-MB-231 and MDA-MB-453 cells were transfected with control or OTUB1 siRNA and treated with 50 ng/mL IFNγ for 24 h. Protein expression was detected and quantified (**A**). Relative mRNA expression was determined through qPCR (**B**). **C** MDA-MB-231 and MDA-MB-453 cells were transfected with control or OTUB1 siRNA and treated with 20 µg/mL cycloheximide (CHX) and 50 ng/mL IFNγ. **D** Representative images for ubiquitinated p27 in co-transfected cells with HA-Ub and either control or OTUB1 siRNA with 0.5 μM MG132 and 50 ng/mL IFNγ. Error bars represent mean ± SEM. *P < 0.01 compared to IFNγ-treated control siRNA.

To explore the potential relationship between OTUB1 and IFNγ-induced p27 expression, we transfected OTUB1 siRNA in MDA -MB-231 and MDA-MB-453 cells. OTUB1 knockdown in both cell lines significantly reduced IFNγ-induced p27 expression without affecting mRNA levels (Fig. 3A, B). Co-treatment with cycloheximide, a protein synthesis inhibitor, and IFNγ increased p27 degradation in a time-dependent manner (Fig. 3C). Notably, OTUB1 knockdown in MDA-MB-231 and MDA-MB-453 cells induced rapid p27 degradation at all tested times (0–6 h) and even under basal conditions (0 h) compared to the control siRNA group (Fig. 3C). To assess the impact of OTUB1 on ubiquitin-mediated p27 protein degradation following IFNγ treatment, we conducted a ubiquitination assay. OTUB1 knockdown increased the levels of ubiquitinated p27 in MDA-MB-231 and MDA-MB-453 cells compared to the control siRNA group (Fig. 3D). These findings suggest that OTUB1 modulates p27 protein stability by regulating the ubiquitin-proteasome system.

### IFNγ−mediated OTUB1 phosphorylation at Tyr26 plays a critical role in stabilizing p27

**Fig. 4:**
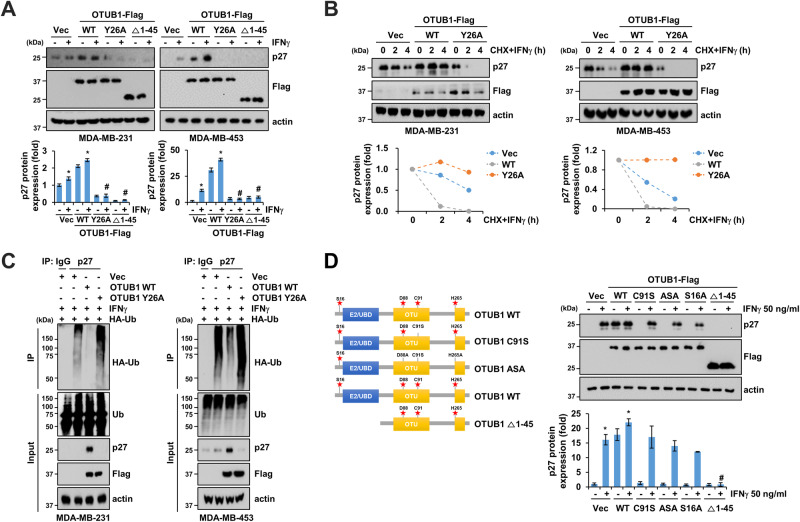
Phosphorylation of OTUB1 at Tyr26 stabilizes p27 expression. **A** MDA-MB-231 and MDA-MB-453 cells were transfected with vector, wild type (WT), Y26A, or $\Delta$1-45 of OTUB1 and treated with or without 50 ng/mL IFNγ. **B** CHX and IFNγ treatment in MDA-MB231 and MDA-MB-453 cells following vector, OTUB1 WT, or OTUB1 Y26A transfection. Representative western blotting image and quantified p27 protein turnover half-life. **C** Representative images for ubiquitinated p27 in MDA-MB-231 and MDA-MB-453 cells transfected with indicated plasmids with combinations of 0.5 μM MG132 and 50 ng/mL IFNγ. **D** Schematic diagram of human OTUB1 domains and strategy to engineer the deletion mutant and point mutants (left). MDA-MB-231 cells were transfected with indicated plasmids in the presence or absence of IFNγ. Error bars represent mean ± SEM. *P < 0.01 compared to control. ^#^*P* < 0.01 compared to control siRNA-transfected cells following IFNγ treatment.

Observing the increased phosphorylation of OTUB1 and p27 expression following IFNγ treatment led us to hypothesize that OTUB1 Tyr26 phosphorylation could regulate p27 stability. To test this hypothesis, we generated OTUB1 mutants, including Tyr26 replaced by alanine (Y26A) and NH2-terminal deletion of residues 1–45 (Δ1–45). Overexpressing wild-type (WT) OTUB1 significantly increased IFNγ-induced p27 expression in both MDA-MB-231 and MDA-MB-453 cells (Fig. 4A). Conversely, the two mutants (Y26A and Δ1–45) of OTUB1 completely suppressed p27 expression in IFNγ-treated cells (Fig. 4A). OTUB1 WT inhibited p27 degradation in response to cycloheximide, whereas the Y26A mutation promoted p27 degradation in both MDA-MB-231 and MDA-MB-453 cells (Fig. 4B). The OTUB1 Y26A mutant strongly abrogated p27 ubiquitination under IFNγ treatment conditions compared to OTUB1 WT in both cell lines (Fig. 4C).

We then investigated whether the canonical action of OTUB1 was linked to p27 expression using OTUB1 catalytic mutants (C91S and D88A/C91S/H265A [ASA]). MDA-MB-231 cells transfected with OTUB1 C91S and ASA showed sustained p27 expression following IFNγ treatment compared to the corresponding controls (Fig. 4D). Additionally, we assessed the effects of OTUB1 phosphorylation at Ser16 on p27 expression. Surprisingly, IFNγ-induced p27 expression remained unchanged with the OTUB1 S16A mutant, similar to the expression in OTUB1 catalytic mutant-transfected cells. Furthermore, p27 expression was fully disrupted by the Δ1–45 (Fig. 4D). These findings suggest that OTUB1 Y26A could serve as a specific and potent site controlling IFNγ-induced p27 expression, emphasizing the crucial role of OTUB1 phosphorylation at Tyr26 in IFNγ-mediated p27 stabilization through deubiquitination.

### OTUB1 downregulation and OTUB1 Y26A mutant eliminates IFNγ-mediated G0/G1 cell cycle arrest

**Fig. 5:**
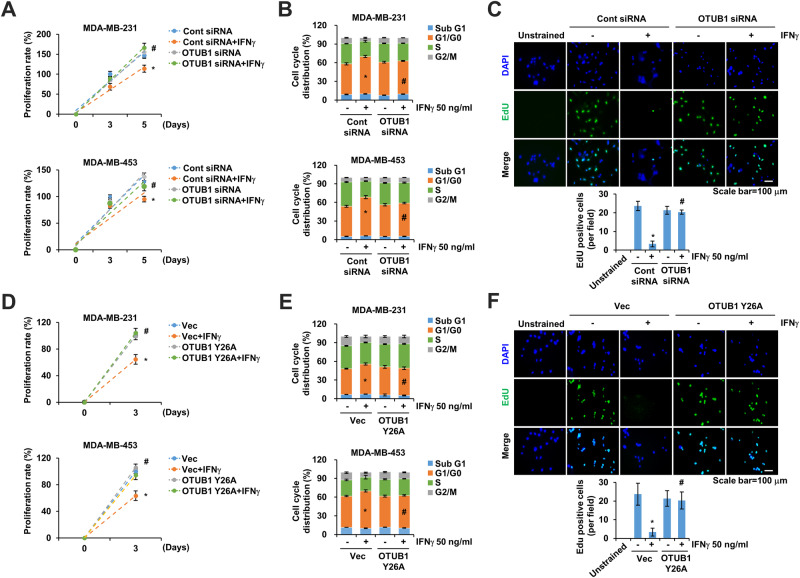
OTUB1 downregulation inhibits IFNγ-induced cell cycle arrest. **A**–**F** OTUB1 was silenced (**A**–**C**) or overexpressed (**D**–**F**) in MDA-MB-231 and MDA-MB-453 cells. Cells were treated with IFNγ for 3 d. Cell viability was verified using MTT assay at indicated time points (**A**, **D**). The cell cycle was determined using FACS analysis (**B**, **E**). EdU-stained cells were observed under a fluorescence microscope at 20× magnification and counted (**C**, **F**). *P < 0.01 compared to control. ^#^*P* < 0.01 compared to control siRNA- or vector-transfected cells following IFNγ treatment.

To elucidate the biological function of OTUB1 in IFNγ-induced cell cycle arrest, we assessed its effects on cell proliferation and cell cycle distribution. OTUB1 knockdown in MDA-MB-231 and MDA-MB-453 cells eliminated the IFNγ-induced inhibition of cell proliferation on 6 day (Fig. 5A). OTUB1 knockdown did not affect cell cycle distribution in the absence of IFNγ. However, IFNγ-induced G0/G1 arrest was inhibited by OTUB1 knockdown (Fig. 5B). Additionally, EdU-labeled proliferating cells persisted in OTUB1 knockdown cells upon IFNγ treatment, whereas the number of labeled cells decreased in the IFNγ control group (Fig. 5C). Similarly, the OTUB1 Y26A mutant transfected into MDA-MB-231 and MDA-MB-453 cells nullified the IFNγ-mediated inhibition of cell proliferation and G0/G1 cell cycle arrest (Fig. 5D, E). Furthermore, OTUB1 Y26A mutant-transfected cells markedly recovered Edu-labeled cell numbers after IFNγ treatment (Fig. 5F).

### E2 enzymes are associated with OTUB1-mediated p27 expression

**Fig. 6:**
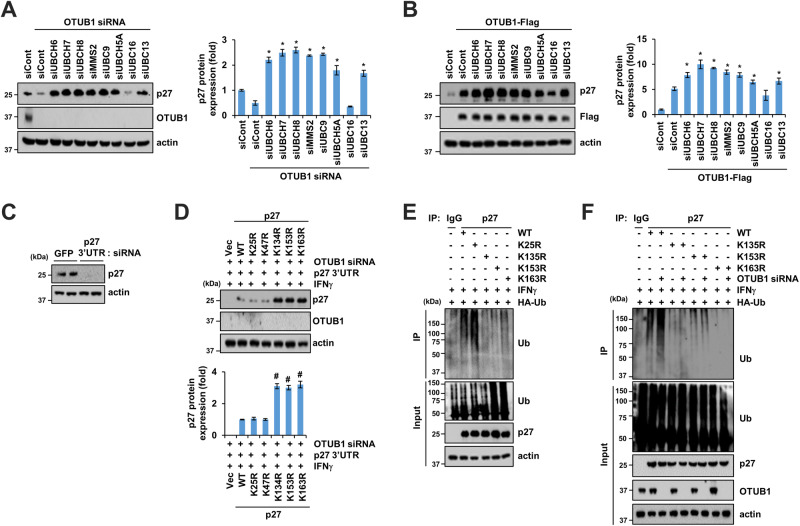
Identification of E2 enzymes and ubiquitination sites of p27 in OTUB1-mediated p27 destabilization. **A**, **B** MDA-MB-231 cells were co-transfected with OTUB1 siRNA (**A**) or Flag-tagged OTUB1 WT (**B**) and eight types of E2 siRNA. **C**, **D** MDA-MB-231 cells were co-transfected with siRNA and indicated plasmids. The knockdown efficacy of endogenous p27 expression was examined by 3′UTR siRNA of p27 (**C**). **E**, **F** Representative images for ubiquitinated p27 in MDA-MB-231 cells transfected with indicated plasmids (**E**) or co-transfected with OTUB siRNA (**F**). *P < 0.01 compared to OTUB1 siRNA- or OTUB1-flag-transfected cells. ^#^*P* < 0.01 compared with p24-transfected cells following IFNγ treatment.

We identified the E2 enzymes involved in p27 deubiquitination. Co-transfection of OTUB1 siRNA with E2 enzymes siRNA (UBCH6, UBCH7, UBCH8, MMS2, UBC9, UBCH5A, and UBC13), except UBC16, resulted in increased p27 expression in MDA-MB-231 cells compared to cells transfected with OTUB1 siRNA alone (Fig. 6A). Similarly, co-transfection of OTUB1 WT with E2 enzymes siRNA strongly induced higher p27 expression in MDA-MB-231 cells than in the corresponding controls (Fig. 6B). These findings suggest that OTUB1-mediated p27 expression may be facilitated by E2 enzymes, such as UBCH6, UBCH7, UBCH8, MMS2, UBC9, UBCH5A, and UBC13.

To investigate the mechanism of OTUB1-mediated p27 ubiquitination, lysine mutants were transfected into MDA-MB-231 cells. 3′UTR p27 siRNA were co-transfected to target endogenous p27 expression (Fig. 6C). K134, K153, and K163 mutations increased exogenous p27 expression, but other mutations such as K25 and K47 did not (Fig. 6D). Consequently, the OTUB1 siRNA-mediated decrease in p27 expression was dramatically rescued by K134, K153, and K163 mutations (Fig. 6E). These mutations completely inhibited p27 ubiquitination, while the K25 mutation did not (Fig. 6F). Increased p27 ubiquitination in OTUB1 siRNA-transfected cells was also decisively blocked by the K134, K153, and K163 mutations in p27 (Fig. 6F). These data suggest that K134, K153, and K163 are required for p27 ubiquitination upon IFNγ stimulation.

### Src activates OTUB1 phosphorylation and p27 expression

**Fig. 7:**
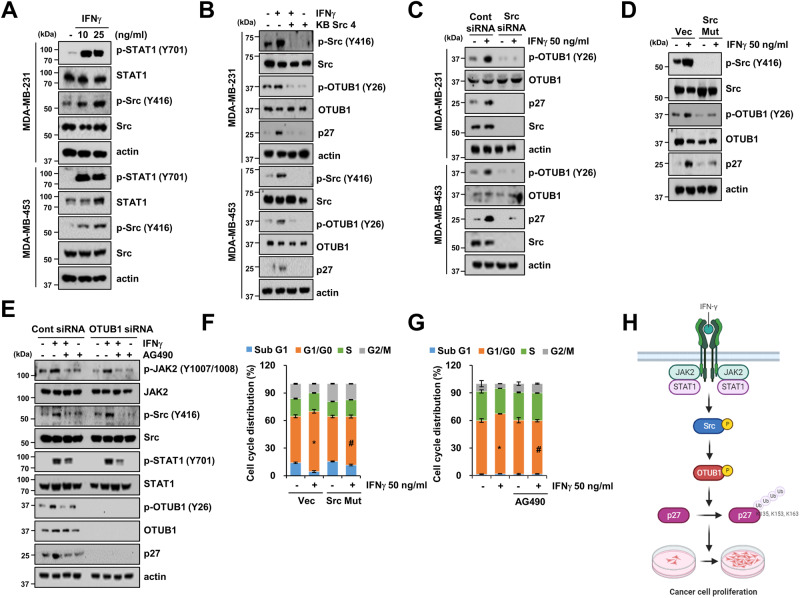
Phosphorylation of OTUB1 Tyr26 is induced by the Jak-Src signaling pathway. **A** Protein expressions of MDA-MB-231 and MDA-MB-453 cells in response to IFNγ treatment. **B** Cells were treated with Src inhibitor (KB Src 4) in the presence or absence of IFNγ. **C** Cells were transfected with either control or Src siRNA and treated with IFNγ for 30 h. **D**, **F** Cells were transfected with either control or Src mutant (Src Mut) plasmid and treated with IFNγ for 30 h. **E**, **G** MDA-MB-231 cells were transfected with either control or OTUB1 siRNA and with IFNγ or JAK2 inhibitor (AG490) alone or in combination with both. Protein expression and cell cycle were determined using western blotting and FACS analysis (**D–G**)**. H** Scheme showing an important role of OTUB1 in IFNγ-mediated cancer cell arrest through p27 upregulation. Created with BioRender.com. *P < 0.01 compared to control. ^#^*P* < 0.01 compared with IFNγ.

Our previous findings demonstrated that Src contributes to the phosphorylation of the Tyr26 residue of OTUB1 [2]. Building on this observation, we aimed to determine whether Src acts as a crucial upstream regulator of OTUB1-mediated p27 expression induced by IFNγ. We observed a dose-dependent increase in p-STAT1 and p-Src expression in IFNγ-treated MDA-MB-231 and MDA-MB-453 cancer cells (Fig. 7A). Inhibition and knockdown of Src, using an Src inhibitor (KB Src 4) and Src siRNA, respectively, remarkably suppressed IFNγ-induced p-OTUB1 at Tyr26 and p27 (Fig. 7B, C). The Src mutation also blocked the expression of these proteins in MDA-MB-231 cells (Fig. 7D). We further explored the impact of IFNγ on upstream signaling involved in regulating OTUB1-p27 expression. Treatment with the JAK2-specific inhibitor AG490 dramatically suppressed the expression of p-Src, p-OTUB1, and p27 upon IFNγ treatment (Fig. 7E). However, p-JAK and p-STAT1 levels did not exhibit obvious differences after OTUB1 knockdown. Moreover, Src mutation and JAK2 inhibition abrogated IFNγ-induced G0/G1 cell cycle arrest in MDA-MB-231 cells (Fig. 7F, G). These data indicate that IFNγ promotes cell cycle arrest in breast cancer cells through the JAK2-Src-OTUB1-p27 signaling pathway.

## Discussion

Previous studies have highlighted the role of IFNγ in tumor progression and immunity, showing cell-type and context-dependent effects [20]. In breast cancer, IFNγ upregulates p27 expression, contributing to cell cycle arrest and cellular senescence [21]. Herein, among various cytokines, IFNγ strongly induced both OTUB1 Tyr26 and p27 expression, whereas no remarkable correlation was observed between OTUB1 Ser16 and p27 expression induced by IFNγ. Notably, OTUB1 exhibited a significantly stronger effect on p27 expression than the other 10 DUBs examined in this study. This observation prompted a detailed exploration of the molecular interplay between OTUB1 and p27 in cancer cells under IFNγ-stimulated conditions. Our gain-of-function and loss-of-function studies unequivocally established that OTUB1 positively regulates p27 protein expression at the post-translational level. Furthermore, OTUB1 Tyr26 controls p27 protein stability and ubiquitination, proposing it as a novel target substrate for OTUB1.

Previous reports have suggested that E2 enzymes play a direct role in the non-canonical action of OTUB1 [2, 22]. OTUB1 deletion substantially degrades E2-conjugating enzymes, including those from the UBC and UBE families, by directly binding to them [23]. We identified specific E2 enzymes associated with OTUB1-mediated p27 expression. Our findings implicate E2 enzymes such as UBCH6, UBCH7, UBCH8, MMS2, UBC9, UBCH5A, and UBC13 in the regulation of OTUB1-mediated p27 expression. Considering the presence of approximately 40 E2 enzymes in humans, further studies are essential to determine the individual contributions of these ligases to p27 expression through OTUB1 deubiquitination.

The JAK2-STAT1 signaling pathway is a well-established universal signal transduction cascade activated by cytokines, including IFNγ [24]. While a previous study on primary astrocytes reported a negative correlation between OTUB1 and JAK, with OTUB1 inhibiting IFNγ-induced activation of JAK2-STAT1 signaling [25], our observations differ. In our study, a JAK2 inhibitor (AG490) attenuated the phosphorylation of OTUB1 (Tyr26) and p27 expression, with no impact from OTUB1, suggesting that JAK2 might serve as an upstream factor activating OTUB1-mediated p27 expression. This discrepancy highlights potential variations in IFNγ-induced signaling pathways across different cell types. As previously demonstrated in renal cancer cells, the non-receptor tyrosine kinase Src mediates OTUB1 Tyr26 phosphorylation [2]. However, the molecular mechanisms and biological functions of Src in IFNγ-induced cell cycle arrest remain poorly understood. Our current study reveals Src as a crucial component for the phosphorylation of OTUB1 (Tyr26) and p27, leading to cell cycle arrest in breast cancer cells. Notably, Src phosphorylation was inhibited by a JAK2 inhibitor (AG490). These findings indicate that IFNγ-induced p27 expression occurs through the JAK-Src-OTUB1 signaling pathway, presenting a potential novel target for controlling cell cycle arrest in cancer cells (Fig. 7H).

In conclusion, our study highlights the significant role of OTUB1 phosphorylation at Tyr26 in cancer cell cycle arrest by stabilizing p27 expression. The JAK-Src signaling pathway appears intricately linked to the regulation of OTUB1-mediated p27 stability. Consequently, our findings propose that targeting OTUB1 could hold therapeutic promise for breast cancer.

## Materials and methods

### Cell culture and reagents

MDA-MB-231, MDA-MB-453, Caki, HCT116, and A549 cells were cultured in Dulbecco’s modified Eagle’s medium (Welgene, Gyeongsan, Korea) supplemented with 10% fetal bovine serum and 1% antibiotics (Thermo Fisher Scientific, Waltham, MA, USA). The cells were maintained at 37 °C in a humidified atmosphere containing 5% CO_2_. Before stimulation with IFNγ (#258-IF-100, R&D Systems, Minneapolis, MN, USA), Tweak (#1090-TW-02, R&D Systems), LPS (#L4391, Sigma-Aldrich, St. Louis, MO, USA), TNF-α (#210-TA-020, R&D Systems), and TGF-β (#240-B-002, R&D Systems), cells were serum-starved overnight.

### 3-(4,5)-Dimethylthiazo(-2-y1)-2,5-diphenytetrazolium bromide (MTT) assay

Cells were seeded in 96-well plates and treated with 25–100 µg/mL IFNγ for 3 d. Control groups were treated with an equivalent amount of phosphate-buffered saline (PBS). After treatment, cells were incubated with 0.2 mg/mL MTT solution (Welgene) at 37 °C for 3 h. Subsequently, the solution was removed, and the resulting formazan was dissolved in isopropyl alcohol. Absorbance was measured at 595 nm.

### Cell cycle analysis

The cells were harvested and fixed in 95% ethanol at 4 °C for 3 h. After removing the fixation solution, RNase was added to sodium citrate buffer at 37 °C for 30 min. Subsequently, 50 µg/mL propidium iodide was added, and the samples were analyzed using a BD Accuri C6 Flow Cytometer (BD Biosciences, San Jose, CA, USA). Cell cycle phases were determined as percentages of the total cell population.

### Cell transfections

Various deubiquitinase (DUB) siRNAs and plasmids were obtained from Bioneer (Daejeon, Korea) as previously described [2]. For siRNA transfection, cells were transfected with 10 nM control or target siRNA using Lipofectamine RNAiMAX (Thermo Fisher Scientific) in Opti-MEM (Gibco). For plasmid transfection, cells were transfected with 1 μg plasmid DNA using Lipofectamine™ 2000 reagent (Thermo Fisher Scientific). The medium was replaced with a complete growth medium 7 h after transfection.

### 5-ethynyl 2′-deoxyuridine (EdU) staining

EdU staining was performed using the Click-iT™ EdU Cell Proliferation Kit for Imaging (C10337; Thermo Fisher Scientific) following the manufacturer’s protocol. MDA-MB-231 and MDA-MB-453 breast cancer cells were cultured on chamber slides, incubated for 24 h with 10 µM EdU, and fixed in 4% paraformaldehyde at room temperature for 15 min. Subsequently, cells were permeabilized with 0.5% triton at room temperature for 20 min, washed with 3% BSA in PBS, and stained with an EdU staining cocktail containing Alexa Fluor 488 dye for 30 min. The fluorescently stained cells were photographed using a fluorescence microscope (Carl Zeiss, Jena, Germany). EdU-stained cells were quantified from randomly selected fields in three independent biological experiments.

### Western blot analysis

Western blotting was performed as previously described [26]. Briefly, cell lysates were separated using 8%–14% sodium dodecyl sulfate-polyacrylamide gel electrophoresis (SDS-PAGE) and transferred to nitrocellulose membranes. The following antibodies were used: p27 (sc-1641), p21 (sc-6246), Ub (sc-8017), OTUB1 (sc-130458), CDK1 (sc-53219), CDK2 (sc-163), CDK4 (sc-260), CDK6 (sc-117), Cyclin A (sc-751), Cyclin D (sc-450), Cyclin E (sc-247), Cyclin B (sc-245), p-Rb (sc-377528), and Rb (sc-74532) from Santa Cruz Biotechnology (Santa Cruz, CA, USA); STAT1 (9172), p-STAT1 (9167), Src (8056), and p-Src (2101) from Cell Signaling Technology (Berkeley, CA, USA); Flag (F1804) and β-actin (A5441) from Sigma-Aldrich. After incubation with horseradish peroxidase-conjugated anti-mouse or anti-rabbit secondary antibodies for 1 h at room temperature, signals were captured and quantified using an iBright CL750 imaging system (Invitrogen, Carlsbad, CA, USA).

### Quantitative real-time PCR (qPCR)

Total RNA isolation, complementary DNA (cDNA) synthesis, and qPCR were performed as previously described [27]. mRNA amplification was assessed using the Thermal Cycler Dice® Real-Time System III (Takara Bio Inc., Shiga, Japan) and determined using the 2^−ΔΔCt^ method. The primers used for p27 and actin amplification were as follows: p27 (forward) 5′-TGTCTTGGAGGAGGATCGTCC-3′, (reverse) 5′-CGGCTCATGGGCGACTTC-3′; actin (forward) 5′-TGGGGTGTTGAAGGTCTC-3′, (reverse) 5′-CTACAATGAGCTGCGTGTG-3′.

### Ubiquitination assay

The ubiquitination assay was performed as previously described [2]. Briefly, cell pellets were suspended in PBS containing 10 mM N-ethylmaleimide (NEM) (EMD Millipore, Darmstadt, Germany) and 1% SDS, then boiled for 10 min at 95 °C. Lysis buffer (1 mL) containing 5 mM NEM was added to the boiled lysates, followed by centrifugation at 13,000 × *g* for 10 min at 4 °C. The supernatants were incubated overnight with the primary antibody and bound to protein G agarose beads for 2 h. Subsequently, the lysates were centrifuged to remove the supernatant at 13,000 × *g* for 10 min at 4 °C and washed with a washing buffer containing 5 mM NEM. The samples were resuspended in 2X sample buffer and boiled at 95 °C for 10 min. Ubiquitinated signals were detected using an iBright CL750 imaging system (Invitrogen).

### Statistical analysis

Statistical analyses were conducted using SPSS v.20.0 (SPSS Inc., Chicago, IL, USA). Group comparisons were assessed using a two-tailed *t*-test.

### Supplementary information

Supplementary Figure 1

## Data Availability

All data contained in this study are included in the manuscript. If more is needed, it is available from the corresponding author.

## References

[1] Zhou K, Mai H, Zheng S, Cai W, Yang X, Chen Z (2020). OTUB1-mediated deubiquitination of FOXM1 up-regulates ECT-2 to promote tumor progression in renal cell carcinoma. Cell Biosci.

[2] Seo SU, Woo SM, Kim MW, Lee EW. Phosphorylation of OTUB1 at Tyr 26 stabilizes the mTORC1 component. Cell Death Differ. 2023;30:82–93. 10.1038/s41418-022-01047-3 PMC9883261 35927303

[3] Liu T, Jiang L, Tavana O, Gu W (2019). The Deubiquitylase OTUB1 Mediates Ferroptosis via Stabilization of SLC7A11. Cancer Res.

[4] Zhao Y, Ruan J, Li Z, Su X, Chen K, Lin Y (2023). OTUB1 inhibits breast cancer by non-canonically stabilizing CCN6. Clin Transl Med.

[5] Nakada S, Tai I, Panier S, Al-Hakim A, Iemura S, Juang YC (2010). Non-canonical inhibition of DNA damage-dependent ubiquitination by OTUB1. Nature.

[6] Juang YC, Landry MC, Sanches M, Vittal V, Leung CC, Ceccarelli DF (2012). OTUB1 co-opts Lys48-linked ubiquitin recognition to suppress E2 enzyme function. Mol Cell.

[7] Liu X, Deng H, Tang J, Wang Z, Zhu C, Cai X (2022). OTUB1 augments hypoxia signaling via its non-canonical ubiquitination inhibition of HIF-1α during hypoxia adaptation. Cell Death Dis.

[8] Ossina NK, Cannas A, Powers VC, Fitzpatrick PA, Knight JD, Gilbert JR (1997). Interferon-gamma modulates a p53-independent apoptotic pathway and apoptosis-related gene expression. J Biol Chem.

[9] Su Q, Wang F, Dong Z, Chen M, Cao R (2020). IFN‑γ induces apoptosis in human melanocytes by activating the JAK1/STAT1 signaling pathway. Mol Med Rep.

[10] Jorgovanovic D, Song M, Wang L, Zhang Y (2020). Roles of IFN-γ in tumor progression and regression: a review. Biomark Res.

[11] Chen HC, Chou AS, Liu YC, Hsieh CH, Kang CC, Pang ST (2011). Induction of metastatic cancer stem cells from the NK/LAK-resistant floating, but not adherent, subset of the UP-LN1 carcinoma cell line by IFN-γ. Lab Invest.

[12] Lo UG, Bao J, Cen J, Yeh HC, Luo J, Tan W (2019). Interferon-induced IFIT5 promotes epithelial-to-mesenchymal transition leading to renal cancer invasion. Am J Clin Exp Urol.

[13] Li Q, Kawamura K, Ma G, Iwata F, Numasaki M, Suzuki N (2010). Interferon-λ induces G1 phase arrest or apoptosis in oesophageal carcinoma cells and produces anti-tumour effects in combination with anti-cancer agents. Eur J Cancer.

[14] Ni C, Wu P, Zhu X, Ye J, Zhang Z, Chen Z (2013). IFN-γ selectively exerts pro-apoptotic effects on tumor-initiating label-retaining colon cancer cells. Cancer Lett.

[15] Song M, Ping Y, Zhang K, Yang L, Li F, Zhang C (2019). Low-Dose IFNγ Induces Tumor Cell Stemness in Tumor Microenvironment of Non-Small Cell Lung Cancer. Cancer Res.

[16] Matsushita H, Hosoi A, Ueha S, Abe J, Fujieda N, Tomura M (2015). Cytotoxic T Lymphocytes Block Tumor Growth Both by Lytic Activity and IFNγ-Dependent Cell-Cycle ArrestIFNγ-Dependent Cell-Cycle Regulation by CTL Therapy. Cancer Immunol Res.

[17] Kortylewski M, Komyod W, Kauffmann ME, Bosserhoff A, Heinrich PC, Behrmann I (2004). Interferon-gamma-mediated growth regulation of melanoma cells: involvement of STAT1-dependent and STAT1-independent signals. J Invest Dermatol.

[18] Satoh T, Kaida D (2016). Upregulation of p27 cyclin-dependent kinase inhibitor and a C-terminus truncated form of p27 contributes to G1 phase arrest. Sci Rep.

[19] Chen Q, Xie W, Kuhn DJ, Voorhees PM, Lopez-Girona A, Mendy D (2008). Targeting the p27 E3 ligase SCF(Skp2) results in p27- and Skp2-mediated cell-cycle arrest and activation of autophagy. Blood.

[20] Tecalco-Cruz AC, Macías-Silva M, Ramírez-Jarquín JO, Méndez-Ambrosio B (2021). Identification of genes modulated by interferon gamma in breast cancer cells. Biochem Biophys Rep.

[21] Matsushita H, Hosoi A, Ueha S, Abe J, Fujieda N, Tomura M (2015). Cytotoxic T lymphocytes block tumor growth both by lytic activity and IFNγ-dependent cell-cycle arrest. Cancer Immunol Res.

[22] Saldana M, VanderVorst K, Berg AL, Lee H, Carraway KL (2019). Otubain 1: a non-canonical deubiquitinase with an emerging role in cancer. Endocr Relat Cancer.

[23] Pasupala N, Morrow ME, Que LT, Malynn BA, Ma A, Wolberger C (2018). OTUB1 non-catalytically stabilizes the E2 ubiquitin-conjugating enzyme UBE2E1 by preventing its autoubiquitination. J Biol Chem.

[24] Murray PJ (2007). The JAK-STAT signaling pathway: input and output integration. J Immunol.

[25] Wang X, Mulas F, Yi W, Brunn A, Nishanth G, Just S (2019). OTUB1 inhibits CNS autoimmunity by preventing IFN-γ-induced hyperactivation of astrocytes. EMBO J.

[26] Lee SG, Lee E, Chae J (2022). Bioconverted Fruit Extract of Akebia Quinata Exhibits Anti-Obesity Effects in High-Fat Diet-Induced Obese Rats. Nutrients.

[27] Seo SU, Woo SM, Lee SG, Kim MY, Lee HS, Choi YH (2022). BAP1 phosphorylation-mediated Sp1 stabilization plays a critical role in cathepsin K inhibition-induced C-terminal p53-dependent Bax upregulation. Redox Biol.

