# Supplementary figures and images for: Non-canonical deubiquitination of OTUB1 induces IFNγ-mediated cell cycle arrest via regulation of p27 stability
Source: Oncogene. 2024 Apr 25;43(24):1852–60. doi: 10.1038/s41388-024-03042-z (PMC11164677; doi:10.1038/s41388-024-03042-z)

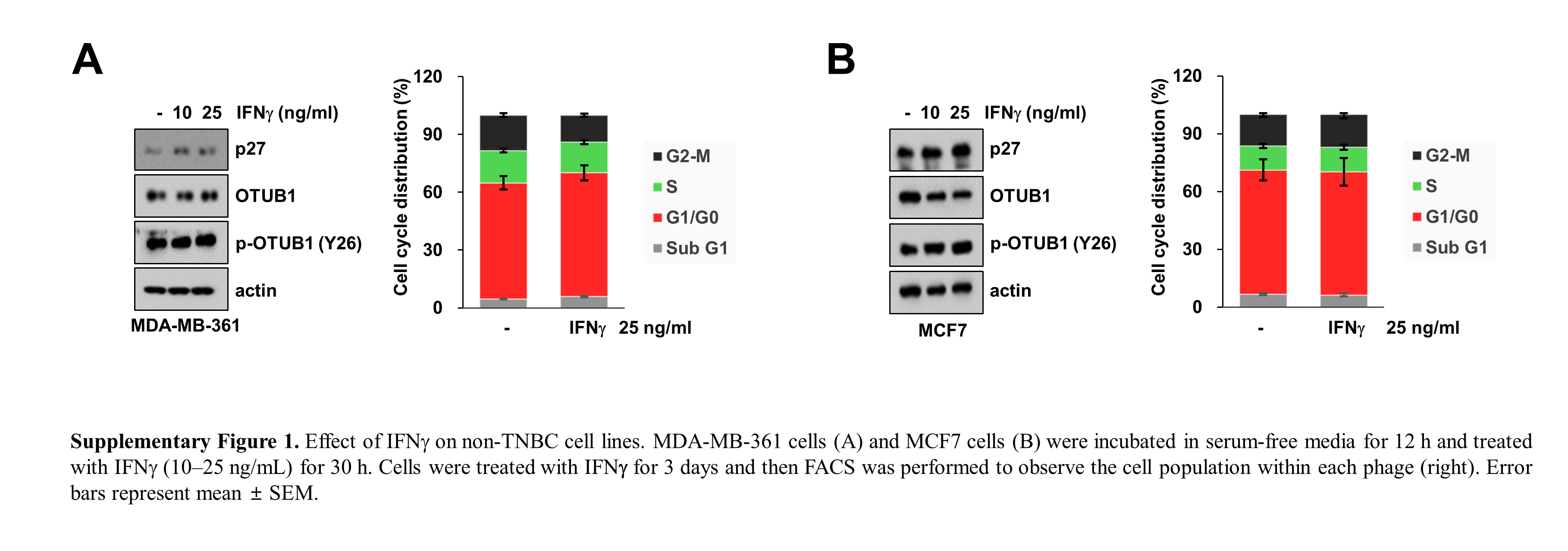

Supplement: Supplementary file 1 — Supplementary Figure 1 [file 41388_2024_3042_MOESM1_ESM.tif]
